# The Pharmacological Implications of Flavopiridol: An Updated Overview

**DOI:** 10.3390/molecules28227530

**Published:** 2023-11-10

**Authors:** Hemant Joshi, Hardeep Singh Tuli, Anuj Ranjan, Abhishek Chauhan, Shafiul Haque, Seema Ramniwas, Gurpreet Kaur Bhatia, Divya Kandari

**Affiliations:** 1 School of Biotechnology, Jawaharlal Nehru University, New Delhi 110067, India; hemantjoshibcas@gmail.com; 2 Department of Bio-Sciences and Technology, Maharishi Markandeshwar Engineering College, Maharishi Markandeshwar (Deemed to Be University), Mullana, Ambala 133207, India; hardeep.biotech@gmail.com; 3 Academy of Biology and Biotechnology, Southern Federal University, Stachki 194/1, Rostov-on-Don 344090, Russia; aranjan@amity.edu; 4 Amity Institute of Environmental Toxicology Safety and Management, Amity University, Sector 125, Noida 201301, India; akchauhan@amity.edu; 5 Research and Scientific Studies Unit, College of Nursing and Allied Health Sciences, Jazan University, Jazan 45142, Saudi Arabia; shafiul.haque@hotmail.com; 6 Gilbert and Rose-Marie Chagoury School of Medicine, Lebanese American University, Beirut 11022801, Lebanon; 7 Centre of Medical and Bio-Allied Health Sciences Research, Ajman University, Ajman 13306, United Arab Emirates; 8 University Centre for Research and Development, University Institute of Pharmaceutical Sciences, Chandigarh University, Gharuan, Mohali 140413, India; seema.ramniwas@gmail.com; 9 Department of Physics, Maharishi Markandeshwar (Deemed to be University), Mullana, Ambala 133207, India; gurpreet1308@gmail.com

**Keywords:** flavopiridol, flavones, anti-metastasis, apoptosis, anti-inflammatory, cell cycle arrest, anti-viral

## Abstract

Flavopiridol is a flavone synthesized from the natural product rohitukine, which is derived from an Indian medicinal plant, namely *Dysoxylum binectariferum Hiern*. A deeper understanding of the biological mechanisms by which such molecules act may allow scientists to develop effective therapeutic strategies against a variety of life-threatening diseases, such as cancer, viruses, fungal infections, parasites, and neurodegenerative diseases. Mechanistic insight of flavopiridol reveals its potential for kinase inhibitory activity of CDKs (cyclin-dependent kinases) and other kinases, leading to the inhibition of various processes, including cell cycle progression, apoptosis, tumor proliferation, angiogenesis, tumor metastasis, and the inflammation process. The synthetic derivatives of flavopiridol have overcome a few demerits of its parent compound. Moreover, these derivatives have much improved CDK-inhibitory activity and therapeutic abilities for treating severe human diseases. It appears that flavopiridol has potential as a candidate for the formulation of an integrated strategy to combat and alleviate human diseases. This review article aims to unravel the potential therapeutic effectiveness of flavopiridol and its possible mechanism of action.

## 1. Introduction

Plants, the only producers in ecosystems, have influenced humankind and other life systems. Novel and effective therapeutic agents can be developed using bioactive products from plants that can be applicable to various dreadful illnesses, such as cancer and viral, fungal, and neurological diseases [1]. This review emphasizes the therapeutic benefits associated with a compound named flavopiridol (FP). Basically, FP is a flavone and semi-synthetic derivative of the naturally occurring product rohitukine [2]. Rohitukine is a chromone alkaloid found in four different plants: *Dysoxylum binectariferum* Hiern. (stem bark), *Amoora rohituka* (Roxb.) Wight & Arn.-(stem and leaves), *Schumanniophyton problematicum*, and *Schumanniophyton magnificum* [3,4]. Rohitukine is widely used as a medicine on the Indian subcontinent. It has been found in preclinical studies that FP is a potent anti-cancer medication for leukemia, lymphoma, prostate carcinoma, breast cancer, bladder cancer, and liver cancer [5,6,7,8,9]. Flavopiridol performs its anti-cancer function by inhibiting multiple cyclin-dependent kinases (CDKs) [10]. CDK protein kinases belong to the serine/threonine protein kinase family. They regulate cell cycle progression and transcription and are a potential target for developing chemopreventive therapies [11,12]. Several CDKs, including CDK1, 2, 4, and 6, directly participate in cell progression, whereas CDK7, 8, and 9 assist in transcription [2,13].

The enzymatic activities of CDK1, 2, and 4 are suppressed by flavopiridol, which leads to cell cycle arrests in the G1 and G2 phases, preventing them from entering the synthesis (S) and metaphase (M) phases, respectively [14,15,16]. In addition, it inhibits CDK9, a part of the positive transcription elongation factor b (P-TEFb) that leads to the suppression of phosphorylation at the C-terminus of RNA polymerase II, inhibiting transcription [17]. Among other CDK inhibitors, FP was the first to be studied in human trials [18,19,20]. In 1994, clinical studies first evaluated it as a combined therapeutic regimen for acute myeloid and chronic lymphocytic leukemia [21]. Despite the good results in preclinical studies, some unwanted toxicities (i.e., secretory diarrhea, hypotension, and pro-inflammatory syndrome) were found at higher doses of FP in clinical trials [22]. Numerous derivatives of FP were synthesized to overcome this issue by chemical modification to improve its CDK activity, biodistribution, and therapeutic potential [23,24,25].

Flavopiridol has been extensively explored for its pharmacological therapeutic implications over the last few decades. Flavopiridol modulates several signaling pathways associated with tumor proliferation and metastasis, viral replication, fungal infection, and inflammation-associated diseases [26,27,28,29,30]. Even though such bioactive semi-synthetic compounds are available, their use in treating cancer and viral, fungal, and inflammation-associated diseases is yet to be determined. Thus, understanding how a therapeutic agent such as FP acts is essential. This is imperative so that the research community can better comprehend the different signaling mechanisms underlying the onset of disease and develop innovative treatment approaches. A review is provided here, summarizing the many therapeutic applications of FP, as well as the possible molecular mechanisms of its actions.

## 2. Chemistry of Flavopiridol

**Figure 1:**
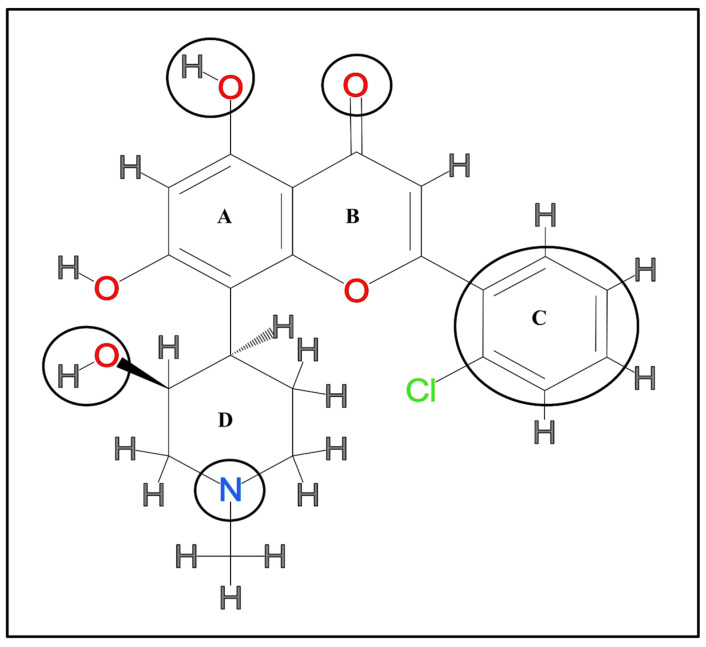
The chemical structure of flavopiridol. A, B, C, and D represent the different aromatic rings present in the structure. The encircled regions mark the functional groups essential to flavopiridol’s functional activity.

Flavopiridol is also known as alvocidib, L86-8275, HL275, and NSC649890. The chemical name of FP is [4,31]-1-benzopyran-4-one] [32]. This flavone is synthetically produced from the natural anti-rheumatic flavonoid rohitukine. Flavopiridol is a yellow crystalline solid that can be identified through different spectroscopic and chromatographic techniques. Its molecular formula is C_21_H_20_ClNO_5_; its melting temperature ranges from 186 °C to 190 °C, and its molecular weight is 401.84 g mol^−1^. Flavopiridol has minimal solubility in water; however, it is soluble in organic solvents, including ethanol, dimethyl sulfoxide, and dimethyl formamide [33]. The synthesis of FP occurs in seven steps of chemical reactions. A detailed version of its synthesis has been reported previously [34]. The crystal structure of FP was determined in complex with the cyclin-dependent kinase2 (CDK2) enzyme, which confers the flavone nucleus of the former bound to the ATP binding site pocket of the latter [35]. It shows CDK-inhibitory activity by containing the D-ring, also known as the 3-hydroxy-1-methylpiperidinyl ring, as shown in Figure 1. In contrast, this ring is absent in two structurally relevant natural flavonoids, quercetin and genistein, that show poor CDK-inhibitory activity [34,36,37].

## 3. Molecular Insights into the Chemopreventive Actions of Flavopiridol

### 3.1. Apoptosis Activation and Cell Cycle Arrest

**Figure 2:**
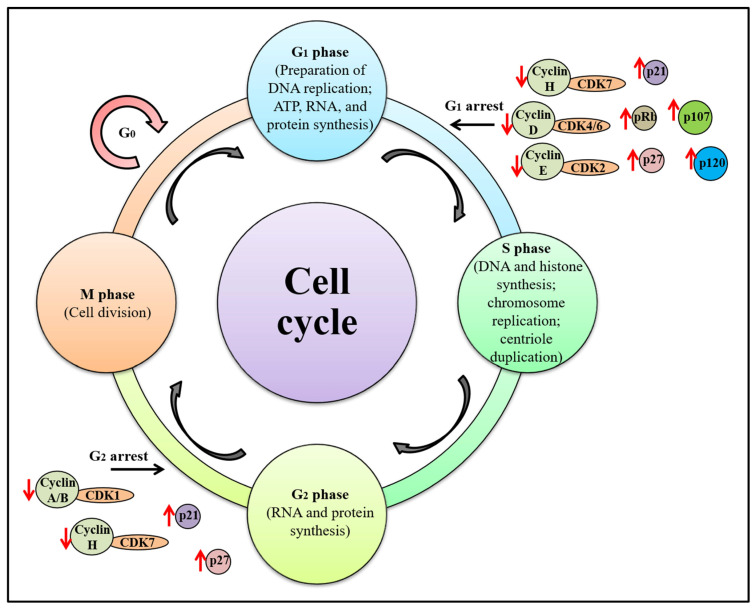
Flavopiridol controls cell cycle progression by targeting different cyclin and CDK proteins of the G1 and G2 phases. The red arrows represent the effect of flavopiridol on the expression, and the arrow directions indicate the increase or decrease in the expression of these proteins.

An important criterion that distinguishes tumor cells from healthy cells and promotes tumor formation is the loss of apoptotic functions [38]. Apoptosis is a programmed cell death (PCD) that involves condensation and disintegration of less compact chromatin, cleavage of DNA while forming ladders, and membrane blebbing associated with phosphatidylserine exposure [39]. It is a strictly organized process regulated mainly by two types of apoptotic proteins: pro-apoptotic proteins and anti-apoptotic proteins [31]. Currently, anti-cancer therapies focus on inhibiting the development and proliferation of tumor cells by targeting apoptotic pathways. In the quest to develop effective anti-cancer therapies, numerous anti-cancer compounds are being used, including FP. Flavopiridol shows anti-cancer activities mainly by directly inhibiting CDK (i.e., CDK1, 2, 4, 6, and 7) through ATP-competitive inhibitions and indirectly minimizing the levels of cyclins (i.e., cyclin D1 and cyclin D3) or CDK inhibitors (p21 and p27). Reduced levels of cyclin D (cyclin D1, cyclin A, and cyclin E) further reduce CDK enzymatic activity, leading to impaired phosphorylation of the pRb, p107, and p120 proteins (Figure 2) [17,40]. 

**Figure 3:**
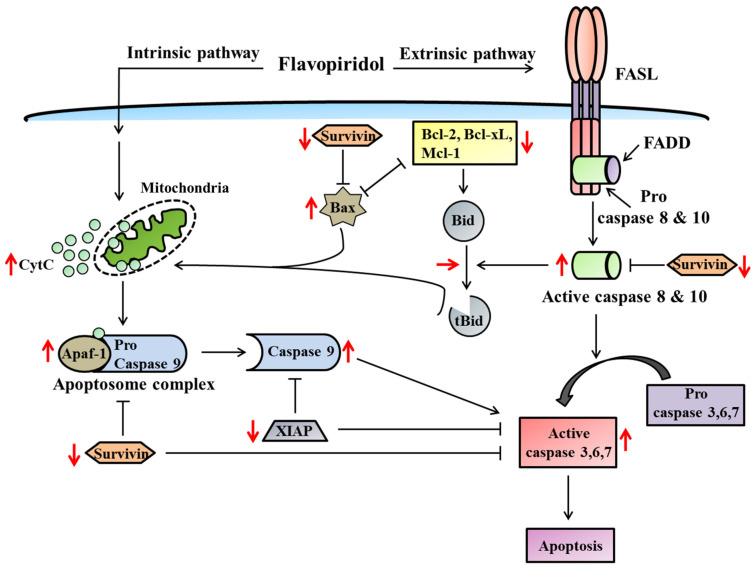
An illustration of the apoptosis induced by flavopiridol. Flavopiridol promotes the apoptosis of tumor cells by targeting different proteins involved in the intrinsic and extrinsic apoptotic pathways. The red arrows represent the effect of flavopiridol on the expression, and the arrow directions indicate the increase or decrease in the expression of the respective proteins.

These hypophosphorylated proteins inhibit various transcription factors (MDM-2, Myc, c-Jun, E2F) mainly responsible for growth arrest and promoting apoptosis [40]. Likewise, FP decreased the levels of p21 and p27, led to the growth arrest at the G1/S phase of the cell cycle, activated caspase 9, increased the ratio of bax/bcl2, and induced apoptosis [41,42]. Smith et al., (2008) have reported that a nanomolar concentration of FP was sufficient to arrest the cell cycle in the G1 and G2 phases, and it decreased expression of cyclin D1, increased expression of p21, and promoted apoptosis by activation of caspase 3 or 7, thereby inhibiting rhabdoid tumor growth [43]. In a study using non-small-cell lung cancer cell lines (NCI-H661 and A549) and an osteosarcoma cell line (U2OS), the pro-apoptotic effect of FP was linked with cdk2/cyclin A inhibition and inactivation of the E2F-1 transcription factor during the late S-phase of the cell cycle, resulting in tumor cell apoptosis [44]. A recent study reported that FP, with CKAP2L (cytoskeleton-associated protein 2-like) depletion, led to the suppression of tumor growth and apoptosis activation in esophageal squamous cell carcinoma [45]. Several studies reported that active proliferating cancerous cells are more sensitive to FP after 24 h of treatment than resting cancerous cells [5,32,40]. Later, it was observed that FP has equal potential to activate cell death in both proliferating and non-proliferating cancer cells after 24 h of treatment [4,46]. Since all solid tumors contain resting cancer cells within hypoxic zones due to a lack of oxygen, FP is highly attractive in this context. This feature of FP gains explicit attention for treating chronic lymphocytic leukemia, where most of the lymphocytes exist in the quiescent stage [4]. In a majority of studies to date, FP-induced tumor cell death has been determined to be independent of p53, decreasing the expression of several anti-apoptotic proteins (i.e., Bcl-X_L_, Bcl-2, XIAP, survivin, and Mcl-1) as shown in Figure 3 [47,48,49,50,51]. 

Survivin is an apoptosis inhibitor in its phosphorylated form and is responsible for abnormal progression of cell cycles, as well as dodging apoptosis. However, FP suppresses the phosphorylation of survivin at threonine 34, resulting in loss of survivin activity and favoring the activation of cell death in MCF-7 (human breast cancer cells) and HeLa (human cervical cancer cells) [52]. In a study using atypical thyroid cancer cells, Pinto et al., (2020) observed that FP inhibits cell proliferation, downregulates Mcl-1, and favors induction of cell cycle arrest [53]. In multiple myeloma cells, FP was found to downregulate the levels of anti-apoptotic proteins (Mcl-1) and the induction of apoptosis. However, overexpressed Mcl-1 multiple myeloma cell lines restrict FP-induced apoptosis [49]. Further, FP mediates apoptosis in tumor cells via caspase-dependent and -independent mechanisms (Figure 3). Caspase-dependent tumor cell death in breast cancer cells (MDA-MB-435), Burkitt’s lymphoma cell line (GA-10), and human myeloid leukemic cells (U937 and HL-60) has been mediated by FP through the induction of classical mitochondrial proteins, including activation of caspases (caspase 3, 8, and 9), DNA laddering, cleavage of PARP (poly ADP-ribose polymerase) and Bid proteins, elevated levels of Bax, decreased expression of Bcl2, and the release of cytochrome C (cytC) (Figure 3) [54,55,56,57]. In lung or esophageal cancer cells, FP, combined with depsipeptide, mediates apoptosis by activating caspase 9 and the mitochondrial release of cytC [58]. Both in vitro and in vivo studies on human cholangiocarcinoma cell lines showed that FP potentiates cell cycle abortion and enhances caspase-dependent cell death [59]. Some researchers reported that FP in combination with CPT11 (a DNA topoisomerase inhibitor) promotes the cleavage of apoptosis inhibitors and activation of cell death in colon cancer cells (Hct116) [60]. In contrast, caspase- and Bcl2-independent cell death occurred in glioma and lung carcinoma tumors by FP via the secretion of mitochondrial apoptosis-inducing factor (AIF) [61,62]. In addition, a study reported that FP promotes cell death in murine glioma cells (GL261) through the induction of caspase-dependent and -independent cell death by the secretion of cytC and AIF from the mitochondria, respectively [63]. Moreover, FP may suppress the activities of the transcription factor NF-κB and make tumor cells more vulnerable to chemotherapy and radiation therapy. Through the phosphorylation of the Akt protein, the NF-κB transcription factor indirectly regulates a variety of cellular events, including cell proliferation, migration, and apoptosis. Studies have demonstrated that FP potentiates apoptosis in human leukemic cell lines in conjunction with proteasome inhibitors and PI3K (phosphatidylinositol-3-kinase) inhibitors by inhibiting the expression of NF-κB transcription factor [50,64]. Furthermore, FP could also perform synergistic effects with cytostatic drugs to induce tumor cell apoptosis throughout the cell cycle by induction of caspase-dependent and -independent mechanisms [65]. Using MKN-74 (human gastric tumor cells) and MCF-7, Motwani et al., (1999) investigated the synergistic effect of FP with paclitaxel to enhance the caspase activity and found that PARP cleavage led to the activation of cell death [66]. Interestingly, FP in synergy with gemcitabine potentiates the activation of caspase-dependent cell death in human gastric, colon, and pancreatic tumors by suppression of the transcriptional activation of the ribonucleotide reductase M2 subunit [67]. In another experimental study, it was demonstrated that FP enhances mitomycin-C-induced apoptosis in MKN-74 (gastric tumor cells) and MDA-MB-468 (breast cancer tumor cells) by inhibiting the activity of protein kinase C [68]. Likewise, irinotecan-induced apoptosis in advanced hepatocellular carcinoma is potentiated by FP [69]. As a collective, these shreds of evidence suggest that FP individually or in synergy with chemopreventive drugs enhances the induction of tumor cell apoptosis via caspase-dependent or -independent mitochondrial pathways by the downregulation of anti-apoptotic proteins, inactivation of cell cycle promoting proteins, and inhibition of tumor cell proliferation.

### 3.2. Anti-Metastatic Effect

**Figure 4:**
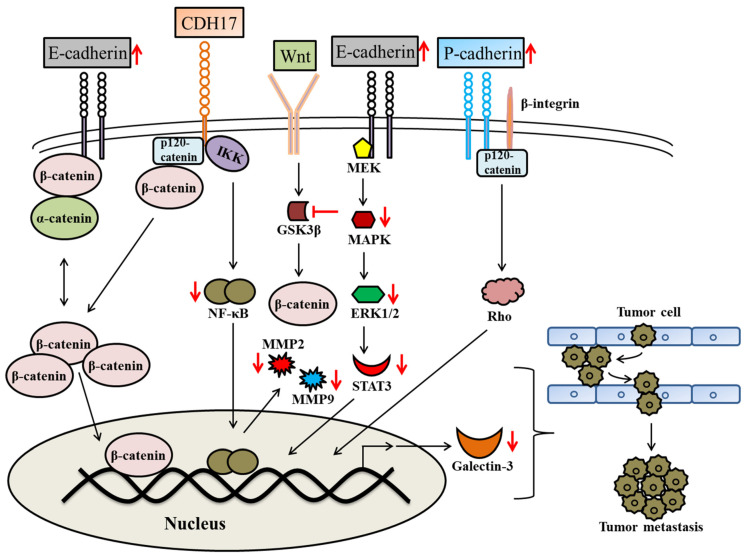
Schematic representation of flavopiridol targeting the regulatory molecular players of tumor metastasis. Flavopiridol mediates the inhibition of tumor metastasis by decreasing MMP proteins’ secretion via inhibition of NF-κB activity, increasing expression of E-cadherin and P-cadherin, and inhibition of wnt signaling through the inhibition of GSK3β. The red arrows represent the actions performed by flavopiridol, whereas the arrow directions indicate the increase or decrease in expression.

Tumor metastasis is one of the fatal characteristics of tumor cells through which they disseminate beyond their original location to surrounding and distant tissues or organs [70]. It occurs in a sequential manner where tumor cells first evade anchorage-dependent cell death (anoikis) by detaching from the extracellular matrix (ECM); second, tumor cells invade across the ECM to gain access to blood circulation through the circulatory system and lymphatic system; third, tumor cells extravasate and arrest at distant sites of the human body; fourth, tumor cells colonize and form micro-metastases by warding off immune attack; and finally, the cells instigate neovascularization to promote tumor growth and establish macro-metastases [71]. An estimated 90% of cancer mortality occurs due to tumor metastasis [38]. Detecting and inhibiting malignant tumor metastasis is a significant challenge that must always be addressed in the modern practice of cancer treatment [70,72]. In order to combat this problem, scientists have found several natural and chemical compounds that inhibit tumor metastasis [73,74,75]. Anti-metastatic agents generally include angiogenesis inhibitors, integrin antagonists, VEGF inhibitors, anti-MMP agents, and receptor tyrosine kinase inhibitors [76,77]. The use of FP as an anti-metastatic agent has been documented for more than three decades in cancer research because it possesses various activities, such as decreased VEGF secretion, anti-angiogenesis activity, anti-invasion activity, and decreased secretion of MMP enzymes (Figure 4) [54,78,79]. Using ovarian carcinoma (OCa-I) as an in vivo tumor model, Mason et al., (2004) determined a significant decrease in metastatic nodules in the lungs after FP treatment [80]. Another group of researchers found that FP-treated human osteosarcoma cells showed anti-metastatic activity, as the inhibition of R-cadherin and induction of P-cadherin expression led to the reduced migration and invasion of cancerous cells [81]. Similarly, it was evidenced that FP decreased expression of galectin-3 and increased expression of E-cadherin in KRAS mutant lung adenocarcinoma cells, which reflects its anti-metastatic effects (Figure 4) [82]. Additionally, in vivo mice experiments confirmed that FP can significantly reduce the ability of human osteosarcoma cells (SJSA-1 and 143B) to metastasize into the lungs [81]. Further, several studies reported that the synergistic interaction between FP and other chemopreventive agents increased their anti-invasive and anti-metastatic activities against metastatic cancer. A recent study investigated FP in conjunction with quisinostat (a histone deacetylase inhibitor), and found that the combination is a potential regimen for treating cutaneous and uveal metastatic melanoma, irrespective of NRAS and KRAS mutations [83]. Likewise, another study reported that a synergistic combination of FP with carfilzomib led to the activation of G2/M cell cycle arrest, decreased levels of X-linked inhibitor of apoptosis (XIAP), and augmented apoptosis. This drug combination exposed to mice with human adrenocortical cancer xenografts provoked a significant decrease in cancerous growth due to increased cleaved caspase and decreased expression of XIAP [84]. Holkova et al., (2011) noted that a bortezomib (proteasome inhibitor) and FP combination is a potential therapeutic option against recurrent or refractory B-cell neoplasms [85]. Similarly, researchers have found that gemcitabine and irinotecan, in combination with FP, possess potentiated anti-metastatic effects that facilitate the treatment of metastatic cancers [86]. In an in vivo breast cancer mice model, FP in combination with sorafenib (Raf inhibitor) had anti-metastatic activity, causing a significant reduction in the lung metastatic tumor. This is possibly due to the decreased Mcl-1 expression and Rb signaling in the cancer cell [87]. In summary, it was concluded that FP itself or combined with other anti-cancerous drugs, has potent tumor metastasis inhibitory activity due to its ability to suppress the exodus and infiltration of tumor cells through numerous mechanisms.

### 3.3. Anti-Angiogenesis

**Figure 5:**
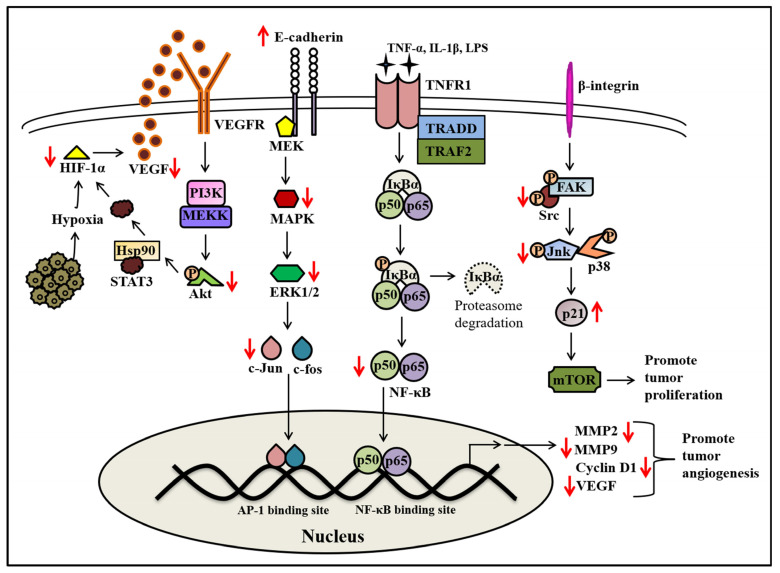
Modulation of molecular targets contributing to tumor angiogenesis by flavopiridol. Flavopiridol inhibits the formation of new blood vessels by targeting different signaling pathways, including decreasing VEGF secretion, suppressing MAPK pathways by inhibiting c-Jun and c-Fos transcription factors, and decreasing the secretion of MMP proteins. The red arrows here represent the actions performed by flavopiridol, whereas the arrow directions indicate the increase or decrease in expression.

Angiogenesis is the process of developing new blood vessels from existing ones. It occurs during menstruation, wound healing, and under various pathological conditions, including tumor growth, arthritic synovium, and proliferative retinopathy [88]. In tumor cells, angiogenesis is mainly responsible for neovascularization through the activation of endothelial cells by tumor-derived cytokines like vascular endothelial growth factor (VEGF), which encourages tumor cells to survive and expand [89]. The most potent angiogenic growth factor, VEGF, is generally released during hypoxic conditions to help tumor cells adapt to this microenvironment, driven by hypoxia-induced factor-1 (HIF-1α) [90]. Inhibition of cancer cell proliferation can be achieved by targeting angiogenic growth factors, oncogenes, membrane receptors, proteases, and signaling transduction factors involved in the neovascularization of tumor cells. Flavopiridol decreased the expression of VEGF mRNA and its consecutive protein in human monocytes under hypoxic environments by reducing VEGF mRNA stability (Figure 5) [91]. Another group of researchers found that FP inhibited the Src transcription factor and downregulated the expression of VEGF [92]. Further, it was evidenced that FP could also decrease *c-fos*-induced VEGFD expression by the inhibition of *fos* promoter activation and PKC [93]. In the studies on GI-LI-N, LAN-5, and ACN (human neuroblastoma cells), researchers demonstrated that FP inhibits the expression of VEGF, which is induced by HIF-1α, picolinic acid, and desferrioxamine [94]. Also, Newcomb et al., (2005) found that the inhibition of angiogenesis by FP in human glioma cell lines (U87MG and T98G) via downregulation of VEGF expression through a decrease in HIF-1α levels occurred even when a proteasome inhibitor was present [78]. Another vital component of the angiogenesis process is matrix metalloproteinase (MMP) enzymes, which are secreted by tumor cells to facilitate cancer cell infiltration and migration through destruction of extracellular matrix components (Figure 5). It was found that FP impaired the release of MMP2 and MMP9 enzymes in breast tumor cells, which eventually caused the suppression of tumor invasion and migration, as observed in a Matrigel invasion assay [54]. Likewise, FP-treated human glioma cells showed decreased secretion of MMP2, were unable to induce the proteolysis of the extracellular matrix, and favored the inhibition of tumor invasion [78]. Takada et al., (2004) noted that FP-mediated inhibition of MMP9 secretion occurred through impaired NF-κB enzymatic activity induced by NF-κB kinase, IκBα kinase, tumor necrosis factor (TNF) receptor 1 (also referred as TNFR1), TNF-receptor-associated factor-2 (TRAF2), and TNF receptor-associated death domain (TRADD) (Figure 5) [79]. Another study observed the anti-angiogenic function of FP in the human colon carcinoma cell line (RKO). It showed a 70% decrease in blood vessel formation using the mouse xenograft Matrigel invasion model [95]. Since human RKO cell lines are devoid of αvβ3- and αvβ5-integrins, this suggests that the FP-mediated anti-angiogenic effect is independent of integrin inhibition. In contrast, Yang et al., (2014) found a synergistic combination of FP with paclitaxel that showed anti-angiogenic effects by decreasing αvβ3 integrin expression in endothelial cells and increasing expression of αvβ3 integrin in ovarian carcinoma cell lines (SKOV3). It was assessed that both in vitro and in vivo anti-angiogenic activity was evaluated using endothelial cell tube formation and tumor microvessel density, respectively [96]. Similarly, a combination of FP with docetaxel in transgenic Gγ/T-15 mice induced apoptosis and decreased angiogenesis, leading to the inhibition of primary and metastatic prostate cancer [97]. Further, authors reported that FP is a better anti-angiogenic molecule than the known angiogenesis inhibitor TNP-470, as observed in a Matrigel invasion model of human colon carcinoma xenografts [98].

Interestingly, Mcferrin et al., (2010) reported another possible mechanism for FP-mediated anti-angiogenic effects. Researchers observed that FP inhibits the activity of CDK9 and vGPCR (Kaposi sarcoma-associated herpesvirus G-protein coupled receptor) in human umbilical vein endothelial cells (HUVECs), which further decreases the secretion of VEGF-A and VEGF-C, leading to a decrease in cell migration and new blood vessel formation [99]. All the pieces of evidence indicated that FP possesses anti-angiogenic effects through numerous mechanisms such as decreasing hypoxic-induced VEGF secretion through decreased HIF-1α expression, decreasing MMP enzyme secretion by inactivation of NF-κB, and inhibition of vGPCR that further inhibits VEGF secretion (Figure 5). Altogether, FP possesses anti-apoptotic and anti-angiogenic activities. Hence, it could be applied to the treatment of different cancers in the future.

### 3.4. Anti-Inflammatory Effects

**Figure 6:**
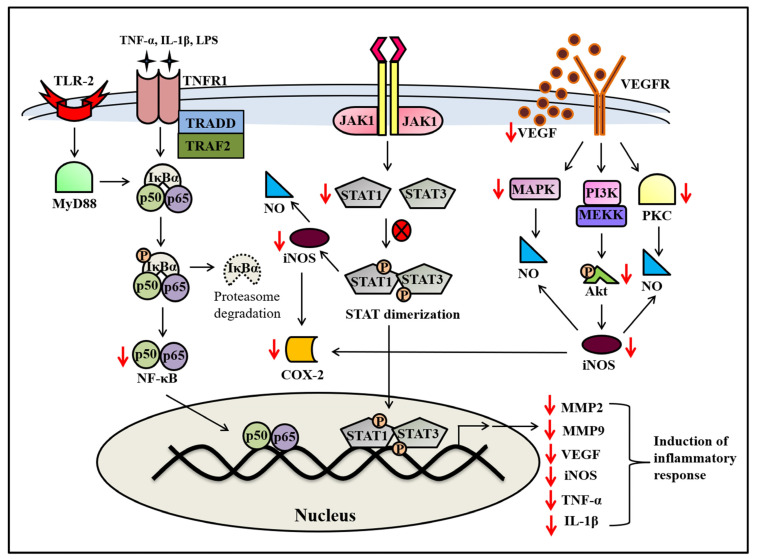
Schematic representation of the anti-inflammatory activity of flavopiridol expression. Flavopiridol exerts its anti-inflammatory effects by targeting proteins of different cell signaling pathways, such as NF-κB, JAK-STAT, MAPK, PI-3K, and PKC. The red arrows here represent the actions performed by flavopiridol, whereas the arrow directions indicate the increase or decrease in expression.

Inflammatory diseases occur when the immune system fails to eliminate organisms or foreign substances, resulting in excessive and prolonged inflammation. This event contributes to the progression and pathogenesis of a broad range of severe inflammation-associated diseases such as cancer, asthma, atherosclerosis, neurological illnesses, and arthritis [100,101]. A study reported the dose-dependent inhibition of inflammatory processes in murine collagen-induced arthritis conditions by FP [102]. One of the marked characteristics of inflammation is massive leukocyte infiltration from the bloodstream into the tissue site via the ability of leukocytes to interact with endothelial cells. FP has the potential to show anti-inflammatory effects by inhibiting the leukocyte extravasation and leukocyte–endothelial cell interaction through the suppression of endothelial cell activation [103]. In support of this fact, it was observed that FP blocked neutrophil infiltration and reduced the cell adhesion molecules (E-selectin, ICAM-1, and VCAM-1) expressions in a concanavalin A-induced murine hepatitis model [103]. Additionally, it was proved that FP-mediated suppression of macrophage infiltration occurs when given through the intrathecal route [104]. Further, it was found that FP abrogates ICAM-1 expression as well as NF-κB mediated gene transcription by impairment of CDK9 activity, not by suppressing the canonical NF-κB activation pathway (i.e., abrogation of IκBα kinase and p65 subunit phosphorylation) [103]. Despite being part of P-TEFb (positive transcriptional elongation factor b), CDK9 has been reported to bind with the cytoplasmic part of glycoprotein (gp130), the receptor for IL-6, a pro-inflammatory cytokine [103]. To strengthen the observation of the previous study, it was observed that FP inhibited IL-6/STAT3 (signal transducer and activation of transcription 3) signaling in hepatocarcinoma (hepG2 cell line) via a JAK/STAT signaling pathway, which is a crucial inflammatory pathway to increase neutrophil survival [105]. Further, it was evidenced that FP suppressed IFN-γ mediated nitric oxide (NO) formation within vascular endothelial cells by reducing expression levels of inducible NO synthase (iNOS), which leads to the inactivation of STAT1 and its downstream molecule IFN-γ responsive factor (IRF1) in JAK/STAT signaling, suggesting an anti-inflammatory effect (Figure 6) [106]. It is imperative to recognize that NO and TNF-α are crucial inflammatory molecules and excess production of them is associated with the pathophysiology of many inflammatory illnesses [107]. Interestingly, Haque et al., (2011) reported that FP decreased the production of pro-inflammatory mediators, including TNF-α and NO, in lipopolysaccharide (LPS)-activated cells [108]. In addition to NF-κB and IκBα kinase, it also inhibits the series of MAPK (mitogen-activated protein kinases), including p38, extracellular signal-regulated kinases 1/2 (ERK1/2), c-Jun N-terminal kinases, and stress-induced protein kinases, in the presence of LPS [108]. Further, it was found that FP-treated inhibition of TNF-α and NO in the presence of lipopolysaccharide occurred by abrogation of MAPK and NF-κB stimulation via the MyD88-(Myeloid differentiation factor 88) dependent TLR2 (Toll-like receptor 2) ligand, which may explain the importance of FP to mitigate the inflammatory response (Figure 6) [108]. Basically, the four major signaling pathways exist, including JAK/STAT, MAPK, PI3K, and NF-κB, which regulate inflammatory signals and promote the survival of leukocytes [101]. FP can inhibit the Akt phosphorylation that suppresses the NF-κB induction and controls different biological events, including migration, proliferation, inflammation, and apoptosis (Figure 6) [57]. Dose- and time-dependent abrogation of TNF-α-induced NF-κB by FP was reported in different cell lines, including human kidney cells (A293), human myeloid leukemic cells (HL60), and human T-lymphocyte cells (Jurkat) [79]. In addition to TNF-α, FP also suppressed NF-κB induced by other molecules, such as inflammatory agents, tumor promoters, and carcinogens [79]. This inhibition occurs through the suppression of Akt phosphorylation, and a canonical NF-κB stimulation cascade results in the suppression of TNF-α-induced NF-κB regulated genes such as MMP9, cyclooxygenase-2, and cyclin D1, indicating the immunomodulatory and anti-inflammatory role of FP as illustrated in Figure 6 [79]. In another study, FP was found to have anti-arthritic effects by inhibiting the production of inflammatory mediators (i.e., iNOS) and extracellular-matrix-degrading genes (i.e., MMP1, 3, 9, and 13), protecting human cartilage explants from the adverse effects of pro-inflammatory cytokines (i.e., IL-1β, TNF-α, and LPS) while maintaining cell survival and function [109]. The in vitro and in vivo studies on the bone remodeling model indicate that FP suppresses RANKL (receptor activator of nuclear kappa B ligand) signaling pathways by suppressing the non-canonical NF-κB activation cascade [110]. Information on RANKL suggests that it is a transmembrane protein of the TNF superfamily and is closely linked to acute and chronic inflammation, which strengthens the role of FP as an anti-inflammatory agent [111,112]. Chang and his colleagues reported the abrogation of inflammatory mediators in FP alone or in combination with PLGA (poly (lactic-co-glycolic acid)) nanoparticle-treated astrocytes through significant inhibition of pro-inflammatory cytokines (i.e., IL-1β, IL-6, and TNF-α) and induction of an anti-inflammatory cytokine (i.e., IL-10) [104,113]. Similar results were obtained in a fungal keratitis mouse model, wherein FP inhibited the inflammation induced in fungal keratitis through the production of the IL-1β, IL-6, and TNF-α cytokines while promoting the production of the IL-10 cytokine [29]. All the evidence indicated herein points towards FP’s ability to inhibit inflammation through a multitude of mechanisms, as discussed above, making it a potential anti-inflammatory agent for future clinical research.

## 4. Efficacy of Flavopiridol as a Therapeutic Agent

The emergence of pathogens that cause life-threatening diseases remains a problem for their treatment and effective disease management and is a significant threat to global public health. Prevention of new infections and their progression to active disease is critical to reducing the burden of the diseases and death caused by them. To address this problem, research is constantly sought for better alternatives to antimicrobial agents due to drug resistance among pathogenic microbes [114]. There is potential for bioactive compounds derived from natural sources to be used to combat drug resistance issues and reduce antibiotic off-target effects [115]. In addition to the anti-cancerous, anti-metastasis, anti-angiogenic, and anti-inflammatory functions of FP, it has also been extensively used as a treatment for different diseases such as viral infections, parasitic infections, and fungal infections caused by several harmful pathogens, as shown in Table 1. It has been observed that it abrogates the replication of different viruses, such as human immunodeficiency virus (HIV), herpes simplex virus (HSV1 and 2), human cytomegalovirus (HCMV), human adenovirus (hAdV5 and hAdV53), and influenza virus A (HINI, H3N2, H7N9) by suppression of mRNA transcription through P-TEFb/CDK9 complex inhibition [26,27,28,116,117]. Similarly, it inhibits the growth of different parasites, including *Leishmania mexicana*, *Toxoplasma gondii*, and *Plasmodium falciparum* [29,118]. Interestingly, it has been noted that FP inhibits fungal growth, biofilm formation, and the adhesion ability of *Aspergillus fumigatus*, decreases inflammation, and induces autophagy [29]. Aside from these, it has several other pharmacological benefits, described in Table 1, and the underlying molecular mechanisms have been proposed. In summary, it was concluded that FP has antimicrobial and other therapeutic benefits that could be used to overcome drug resistance and unnecessary outcomes.

## 5. Derivatives of Flavopiridol

As already stated, FP has CDK-inhibitory activity that abrogates the continuation of the cell cycle by arresting the G1 and G2/M phases. This CDK-inhibitory activity is mainly responsible for anti-cancerous, anti-inflammatory, and other pharmacological applications. However, the significant issues hampering FP’s transition from research to the clinic are non-selective kinase inhibitory activity, low target specificity, moderate target affinity, and bioavailability in biological systems. To overcome all these problems, a wide range of FP derivatives have been synthesized by chemical modifications aiming for better biological and pharmacological functionalities. The structure–activity relationship of FP was mentioned in Figure 1. It has been observed that replacement of the chromone moiety of FP by 4-hydroxy benzofuranone improves the kinase inhibitory activity (CDK1 and CDK2) and selectivity against CDK4 and also enhances the anti-cancerous activity [130]. Thio- and oxo-FP analogs increase the selectivity of FP for CDK1 by discriminating from other kinases. In addition, these FP analogs also possess anti-proliferative activity against different human cancer cell lines [131]. Another piece of research indicates that the D-ring of FP is critical for CDK-inhibitory activity, and olefin analogs of FP have shown selective kinase inhibitory activity against CDK4, as well as tumor growth inhibition in MCF-7 cells [34]. Interestingly, P-276-00 is another derivate of FP that shows selective CDK1, CDK4, and CDK9 inhibitory activity compared to other CDKs. Additionally, it also has anti-tumor activities against 14 human cancer cell lines [132]. Further, it was found that the 2-fluorophenyl analog of FP shows 40-fold more selective inhibition to P-TEFb than other CDKs, leading to the inhibition of HIV-1 Tat transactivation and replication; therefore, it could be used as an anti-HIV1 therapeutic [133]. Voruciclib and IIIM-290 are oral bioavailable FP analogs with selective CDK9 inhibitory activity. Voruciclib has shown anti-tumor activity when combined with other anti-cancer drugs to treat different cancers [24]. Another derivative, IIIM-290, displayed caspase-dependent apoptosis in pancreatic cancer and exhibited anti-tumor activity against a wide range of cancer cell lines [23]. Surprisingly, Ibrahim et al., (2020) have reported that thioether-benzimidazoles analogs of FP by chemical modification at the C-ring led to the inhibition of CDK9 and CDK10. This FP derivative is the only compound that has shown CDK10 inhibitory activity, and it also possesses anti-cancer effects by inhibiting tumor growth in seven cancer cell lines [25]. The CDK-inhibitory activity and anti-cancerous effects of other clinically active FP derivatives are summarized in Table 2 and Table 3, respectively.

## 6. Adverse Effects of Flavopiridol

Flavopiridol has several therapeutic benefits, including anti-cancerous, anti-inflammatory, anti-viral, anti-fungal, and anti-parasitic functions, which have already been described in the previous sections. Despite all these advantages of FP, there is still some dirt in the picture. The significant toxicity concerns associated with using FP are secretory diarrhea and pro-inflammatory syndrome (i.e., tiredness, fever, local tumor pain, and intonation of acute phase reactants) associated with hypotension [19]. Flavopiridol’s effects on in vitro epithelial cell models were assessed to explain its unanticipated toxic effects. Flavopiridol caused chloride secretion in these models, implying that therapeutic antidiarrheal medicines should be used to prevent secretory diarrhea [2]. The maximum tolerated dose (MTD) was increased from 50 mg m^−2^/24 h to 78 mg m^−2^/24 h for three days following prophylactic treatment for diarrhea with cholestyramine and loperamide [2]. As an attempt to understand the pro-inflammatory syndrome, different cytokines were examined in the plasma prior to and following FP administration. It was observed that plasma IL-6 levels significantly increased dose-dependently [19,136]. In the phase I clinical study, it was observed that the MTD of FP was 37.5 mg m^−2^/24 h, and dose-limiting side effects were observed at 53 mg m^−2^/24 h, including fatigue, vomiting, neutropenia, diarrhea, and nausea [2,19]. In addition, non-dose-limiting toxicities, such as anorexia and local tumor pain, were observed. Similarly, in another clinical study, dose-limiting side effects were observed at 40 mg m^−2^/24 h MTD for three days with vomiting and nausea [2]. In a phase II dose escalation study of FP, substantial side effects were observed, including anorexia, fatigue, nausea, tumor pain, dyspnea, diarrhea, and cough [137,138]. In another toxicological study, Wirger et al., (2005) reported that at 1 mg/kg/day of FP over three weeks, different side effects were observed, including leukopenia, lesions in the spleen and bone marrow, and gastrointestinal toxicity [7]. Interestingly, in one study, it was found that FP showed drug resistance, though the mechanism is unknown. Later, it was revealed that FP mediates endoplasmic reticulum (ER) stress-induced autophagy, which is responsible for its resistance [139]. In a recent study, it was found that a majority of FP-treated human prostate cancer cells (DU145) died. However, a small sub-population survived and emerged as FP-resistant cancer cells (DU145^FP^) [140]. DU145^FP^ cancer cells have shown different characteristics, including slow growth, mitochondrial depolarization, induced abundant anti-apoptotic proteins, and less sensitivity to docetaxel and cisplatin [140]. These FP-resistant cancer cells induce mitochondrial lesions that modulate cell metabolism, motility, and advanced neoplastic growth potential [140].

## 7. Clinical Trials: Results and Experiences

Several clinical trials have demonstrated FP’s effectiveness against many cancers, including leukemia, lymphoma, and breast, prostate, pancreatic, stomach, lung, liver, head and neck tumors [141,142]. It has been shown that FP is safe and effective in patients suffering from different cancers when administered intravenously per day for several weeks [141,142]. Two significant drawbacks to this drug being used alone as an anti-cancer agent in clinical trials are low plasma concentrations due to its binding with serum proteins and unwanted side effects due to its high doses [142]. However, in some cases, this phytochemical can exert anti-cancer activity alone or in combination with some established anti-cancer medications, including vorinostat and gemcitabine hydrochloride in adult solid tumors, cisplatin in ovarian epithelial cancer, bortezomib in B-cell neoplasms, doxorubicin hydrochloride in sarcomas, venetoclax in leukemia, paclitaxel in esophageal cancer, and docetaxel in breast and pancreatic cancer [141,142]. It has been reported that over 60 clinical trials, varying in clinical phases, have been performed with FP; these trials are listed in detail in Table 4. 

## 8. Conclusions and Future Perspectives

The current research on FP has demonstrated its effectiveness in numerous clinical areas. It has been suggested that FP can modulate multiple molecular signaling pathways associated with the development of different cancers, and inflammatory, neurological, viral, fungal, and parasitic diseases. However, future research should explore other therapeutic functions of FP using different omics approaches such as system biology, genomics, epigenomics, transcriptomics, proteomics, and metabolomics. It would be possible to overcome the evolution of cellular resistance by synthesizing FP derivatives to improve the therapeutic efficacy of drugs for targeted diseases. It is possible to increase the effectiveness of the FP manifold by using synergistic approaches with other natural and synthetic anti-cancer medications or drug molecules. Furthermore, the application of FP with engineered nanoparticles, such as polymeric nanoparticles, liposomes, magnetic nanoparticles, exosomes, and metallic nanoparticles, enhanced the drug loading capacity, half-life in biological systems, sustained release, and selective biodistribution. Additionally, the pharmacokinetic and pharmacodynamic profiles were ameliorated over traditional formulations, preventing tumor metastasis and enhancing cancer therapy. Yang et al. (2009) reported that liposomal formulations of FP administered to mice led to reduced systemic clearance, improved plasma concentrations, and increased elimination phase half-life relative to FP alone [143,144]. Moreover, the clinical efficacy of FP with different nanoformulations against different cancers and other targeted diseases must be studied.

## Figures and Tables

**Table 1:** Description of the therapeutic effects of flavopiridol and the proposed mechanisms of action.

S. No.	Therapeutics	Diseases	Mechanisms	Dose	Route	Experimental Models	Refs.
1	Anti-Alzheimer	Alzheimer	Rescue from memory impairment and cell cycle reactivation caused by Aβ1-42 oligomers; Aβ-treated mice had improved long-term memory response	0.5–1 mg/kg	i.p.	CD1 mice	[119]
2	Neuroprotective	Ischaemic stroke	Inhibits the phosphorylation of Rb; increased levels of E2F1; inhibits CDK activation and prevents CA1 neuronal cell death	500 µmol/L 500 µM	i.c.v.	Wistar rats	[120,121]
		Spinal cord injury	Reduces expression of cyclin D1, pRb, CDK4, E2F1, and PCNA; increases expression of endogenous CDK inhibitor p27; reduces levels of galactin-3 and Iba-1, leading to decreased number of Iba-1^+^ microglial cells; increases CC1^+^ oligodendrocytes and white matter myelinated area; abrogates RNA Pol II phosphorylation and induction to promote neuronal survival	1 mg/kg	i.p.	Male Sprague-Dawley rats	[122,123]
		Traumatic brain injury	Increases neuronal survival post DNA damage and inactivated astroglial proliferation, microglial activation, and scar formation by blocking cell cycle progression proteins	250 µM	i.c.v.	Male Sprague-Dawley rats	[124]
3	Anti-vasoproliferative	In-stent restenosis	Anti-proliferative effects were observed in human coronary artery smooth muscle cells (HCASMC) by cell cycle arrest at G_1_/S and G_2_/M phases; increases levels of p21, p27, and p53; abrogation of Rb hyperphosphorylation; reduces apoptosis; prevents neointima formation	0.1 µM, 25 mg/mL	-	HCASMC, human coronary artery endothelial cells, rat	[125]
4	Anti-hepatitis	Concavalin A-induced hepatitis	Inhibits ConA-induced hepatitis and neutrophil infiltration; suppresses TNF-α-induced leukocyte–endothelial cell interaction; abrogates the levels of ICAM-1, VCAM-1, E-selectin, and NF-κB by inhibition of CDK9 activity	44 ng	i.v.	C57BL/6 male mice	[103]
5	Anti-viral	Acquired immunodeficiency syndrome	Inhibits the phosphorylation induced by P-TEFb at the C-terminus region of RNA Pol II large subunit; abrogates Tat transactivation and HIV-1 viral replication	6–12 nM	-	Transfecting 293T cells with the HIV-1_HXB2_ provirus, HIV-1NL4–3 viral particles, Jurkat cells	[26]
		Human-immunodeficiency-virus-associated nephropathy	Inhibition of HIV-1 transcript levels in infected glomerular visceral epithelial cells; improved nephropathy in mouse model	2.5 mg/kg	i.p.	HIV-1 NL4-3 transgenic mouse model	[116]
		Herpes simplex virus 1 infection	Inhibition of P-TEFb/CDK9 complex by blocking the phosphorylation at serine-2 residue on the C-terminus region of RNA Pol II; suppresses the replication of HSV-1 and HSV-2 through inhibition of mRNA transcription	450 nM 30 mg	-	HeLa cells, Wistar rats, BALB/c mice	[27,117]
		Human cytomegalovirus infection	Suppresses the replication of HCMV through inhibition of mRNA transcription by CDK9 inhibition	1–5 µM	-	A549, Vero cells	[27]
		Human adenovirus infection	Suppresses the replication of HAdV5 and HAdV53 through inhibition of mRNA transcription by CDK9 inhibition; decreases expression of an early gene of adenovirus *E1A*	1–10 µM	-	A549, Vero cells	[27]
		Influenza A virus infection	Suppresses the replication of H1N1, H3N2, and H7N9 through inhibition of mRNA transcription by CDK9 inhibition	0.59 µM 0.70 µM 0.24 µM	-	A549 cells	[28]
6	Anti-fungal	Fungal keratitis	Downregulation of IL-1β, IL-6, and TNF-α expression and induction of IL-10 expression; increases expression of LC3, Beclin-1, and Atg7 proteins; promotes the phagocytosis of RAW264.7 cells; suppresses biofilm formation, growth, and attachment of *Aspergillus fumigatus*; decreases inflammation in fungal keratitis disease by inducing autophagy	5 µM	s.c.i	RAW 264.7 cells, C57BL/6 female mice	[29]
		Aspergillosis	Acts as a non-competitive inhibitor of UDP-galactopyranose mutase (UGM) to treat *Aspergillus fumigatus* infection	200 µM	-	AfUGM	[118]
7	Anti-leishmanial	Leishmaniasis	Inhibition of CRK3 kinase; inhibition of in vitro growth of *Leishmania Mexicana* promastigotes; suppresses cell cycle progression at G_2_ and G_2_/M phase	2.5 µM	-	*Leishmania Mexicana* promastigotes	[126]
8	Anti-parasitic	*Toxoplasma gondii* infection	Inhibition of TgCRK9 kinase activity led to the abrogation of RNA pol II dependent transcription elongation; inhibition of parasite multiplication and proliferation	4 nM, 8 nM	-	HFF cells, *Toxoplasma gondii* culture	[127]
9	Anti-malarial	Malaria	Abrogates the activity of PfPK5 kinase in *Plasmodium falciparum*; inhibition of DNA synthesis	0.06 µM, 2 µM	-	Red blood cells, *Plasmodium falciparum* culture	[128]
10	Anti-diabetic	Diabetes	Inhibition of glycogen phosphorylase a and b enzymes	15.5 µM	-	Rabbit skeletal muscle, A549 cells	[129]
11	Anti-arthritis	Osteoarthritis	Abolishes the activation of iNOS by IL-1β; inhibits a broad range of inflammatory mediators; inhibits the induction of MMP1,3, 9, and 13; protects the cartilage from the harmful effects of pro-inflammatory cytokines	300 nM	-	Human chondrocytes, human cartilage explants	[109]

**Table 2:** An overview of CDK-inhibitory activity by flavopiridol-based derivatives.

	Derivatives	IC50 (in µM)								Refs.
		CDK							GSK3β	
		1	2	4	6	7	9	10		
1	4,6-Dihydroxy-2-[1-[4-(4-methyl-piperazin-1-yl)-phenyl]-meth-(*E*)-ylidene]-7-(1-methyl-piperidin-4-yl)-benzofuran-3-one	0.80	2.98	1.92	-	-	-	-	1.75	[130]
2	4-[4,6-Dihydroxy-7-(1-methyl-piperidin-4-yl)-3-oxo-3Hbenzofuran-(2*E*)-ylidenemethyl] benzenesulfonamide	0.009	0.03	1.87	-	-	-	-	3.7	
3	4,6-Dihydroxy-7-(1-methyl-piperidin-4-yl)-2-[1-(4-nitrophenyl)-meth-(*E*)-ylidene]-benzofuran-3-one	0.06	0.31	2.21	-	-	-	-	4.8	
4	2-[1-(2-Chloro-phenyl)-meth-(*E*)-ylidene]-4,6-dihydroxy-7-(1-methyl-piperidin-4-yl)-benzofuran-3-one	0.60	3.97	25.25	-	-	-	-	10	
5	4,6-Dihydroxy-7-(1-methyl-piperidin-4-yl)-2-[1-phenylmeth-(*E*)-ylidene]-benzofuran-3-one	0.11	1.28	4.41	-	-	-	-	4.2	
	R_1_-*N*-[2-(2-Chlorophenyl)-5,7-dihydroxy-4-oxo-4Hchromen-8-yl]-R_2_									[134]
6	R_2_ = benzamide	-	91	-	-	-	-	-	-	
7	R_2_ = 4-methylbenzamide	-	90	-	-	-	-	-	-	
8	R_2_ = 4-methoxybenzamide	-	54	-	-	-	-	-	-	
9	R_1_ = 3,5-Dichloro; R_2_ = 2- hydroxyl benzene sulfonamide	-	94	-	-	-	-	-	-	
	R-5,7-dihydroxy-8-(3-hydroxy-1-methyl-4-piperidinyl)-4*H*-1-benzopyran-4-one									[131]
10	R = (±)-(3*SR*,4*RS*)-2-(Ethylthio)	0.46	3.93	2.06	-	-	-	-	-	
11	R = (3*S*,4*R*)-2-[(2-Chlorophenyl)thio]	0.11	2.10	16.2	-	-	-	-	-	
12	R = (3*S*,4*R*)-2-(2-Chlorophenoxy)	0.13	2.11	6.15	-	-	-	-	-	
13	R = (±)-(3*SR*,4*RS*)-2-(Phenylthio)	0.44	6.59	4.10	-	-	-	-	-	
14	R = (±)-(3*SR*,4*RS*)-2-(tert-Butylthio)	0.08	1.07	2.07	-	-	-	-	-	
15	R = (±)-(3*SR*,4*RS*)-2-[(4,6-Dimethylpyrimidin-2-yl)thio]	6.40	40.4	82.5	-	-	-	-	-	
16	R = (±)-(3*SR*,4*RS*)-2-(Phenylamino)	16.3	>25	>25	-	-	-	-	-	
17	R = (±)-(3*SR*,4*RS*)-2-*N*-Piperidyl	2.50	9.69	3.70	-	-	-	-	-	
	*cis*-5,7-dihydroxy-2-(R_1_)-8-[R_2_-piperidinyl]-1-benzopyran-4-one									[34]
18	R_1_ = 2-chlorophenyl; R_2_ = 4-(3-one-1-methyl)	10	-	8	-	-	-	-	-	
19	R_1_ = 2-chlorophenyl; R_2_ = *cis*-8-2-hydroxycyclohexyl	12	-	31	-	-	-	-	-	
20	R_1_ = 2-chlorophenyl; R_2_ = 4-(3-en)	1.1	-	0.8	-	-	-	-	-	
21	R_1_ = 3-chlorophenyl; R_2_ = 4-(3-en)	1.2	-	2.4	-	-	-	-	-	
22	R_1_ = 4-chlorophenyl; R_2_ = 4-(3-en)	1.7	-	0.55	-	-	-	-	-	
23	R_1_ = 2-florophenyl; R_2_ = 4-(3-en)	2.3	-	1.8	-	-	-	-	-	
24	R_1_ = 2-bromophenyl; R_2_ = 4-(3-en)	0.98	-	0.65	-	-	-	-	-	
25	R_1_ = 2-florophenyl; R_2_ = 4-(3-en)	-	-	2.5	-	-	-	-	-	
26	R_1_ = 2-phenyl; R_2_ = 4-(3-en)	-	-	1.0	-	-	-	-	-	
27	R_1_ = 2, 4-dichlorophenyl; R_2_ = 4-(3-en)	1.7	-	1.2	-	-	-	-	-	
28	R_1_ = 4-pyridyl; R_2_ = 4-(3-en)	-	-	0.8	-	-	-	-	-	
29	R_1_ = cyclohexyl; R_2_ = 4-(3-en)	-	-	7	-	-	-	-	-	
30	2-(2-chlorophenyl)-5,7-dihydroxy-8-((2*R*,3*S*)-2-(hydroxymethyl)-1-methylpyrrolidin-3-yl)-4H-chromen-4-one hydrochloride (P-276-00)	0.079	0.224	0.063	0.396	2.870	0.020	-	2.771	[132]
31	2-(2-Chloro-4-(trifluoromethyl)phenyl)-5,7-dihydroxy-8-((2*R*,3*S*)-2-(hydroxymethyl)-1-methylpyrrolidin-3-yl)-4H-1-benzopyran-4-one (Voruciclib)	0.0054	-	0.0039	0.0029	-	0.0017	-	-	[135]
32	2-(2,6-dichlorophenyl)-5,7-dihydroxy-8-[(3*S*,4*R*)-3-hydroxy-1-methylpiperidin-4-yl]chromen-4-one (IIIM-290)	0.0049	0.0155	0.0225	0.045	0.711	0.0019	-	-	[23]
33	2-(4-((1H-benzo[d]imidazol-2-yl)thio)phenyl)-5,7-dihydroxy-8-(1-methyl-1,2,3,6-tetrahydropyridin-4-yl)-4H-chromen-4-one	-	0.162	-	-	-	0.008	-	0.117	[25]
34	5,7-Dihydroxy-8-(1-methyl-1,2,3,6-tetrahydro pyridin-4-yl)-2-(4-((1-methyl-1H-benzo[d] imidazol-2-yl)thio)phenyl)-4Hchromen-4-one	-	0.349	-	-	-	0.014	-	0.160	
35	5,7-Dihydroxy-8-(1-methyl-1,2,3,6-tetrahydro pyridin-4-yl)-2-(4-((5-phenyl-1H-imidazol-2-yl)thio) phenyl)-4H-chromen-4-one	-	0.469	-	-	-	0.009	-	0.356	
36	2-(4-((1H-benzo[d]imidazol-2-yl)thio)-2-chloro phenyl)-5,7-dihydroxy-8-(1-methyl-1,2,3,6-tetrahydropyridin-4-yl)-4H-chromen-4-one	-	1.556	-	-	-	0.015	-	0.216	
37	2-(2-Chloro-4-((1-methyl-1H-benzo[d] imidazol-2-yl)thio) phenyl)-5,7-dihydroxy-8-(1-methyl-1,2,3,6-tetrahydropyridin-4-yl)-4H-chromen-4-one	-	1.725	-	-	-	0.064	0.149	0.059	
	2-R-5,7-dihydroxy-8[(3*S*,4*R*)-3-hydroxy-1-methylpiperidin-4-yl]chromen-4-one									[133]
38	R = phenyl	-	0.196	-	-	-	0.009	-	-	
39	R = 3-chlorophenyl	-	0.164	-	-	-	0.004	-	-	
40	R = 4-chlorophenyl	-	0.287	-	-	-	0.012	-	-	
41	R = 2-fluorophenyl	0.123	0.356	-	-	>10	0.003	-	>1	
42	R = 4-fluorophenyl	-	0.129	-	-	-	0.002	-	-	
43	R = 4-bromophenyl	-	0.223	-	-	-	0.005	-	-	
44	R = 4-tert-butylphenyl	-	0.567	-	-	-	0.019	-	-	
45	R = 4-trifluoromethylphenyl	-	0.302	-	-	-	0.019	-	-	
46	R = 4-hydroxyphenyl	-	0.196	-	-	-	0.010	-	-	
47	R = 2-pyridyl	-	0.886	-	-	-	0.011	-	-	
48	R = 3-pyridyl	-	0.247	-	-	-	0.005	-	-	
49	R = 4-pyridyl	-	0.208	-	-	-	0.006	-	-	
50	R = 2-chloro-3-pyridyl	-	0.314	-	-	-	0.013	-	-	
51	R = 5-methylisoxazole	-	0.238	-	-	-	0.020	-	-	
52	R = 3-vinylphenyl	-	0.130	-	-	-	0.010	-	-	
53	R = 4-vinylphenyl	-	0.206	-	-	-	0.010	-	-	
54	R = 4-fluorophenyl	-	0.208	-	-	-	0.006	-	-	
55	R = 2-bromophenyl	-	0.639	-	-	-	0.005	-	-	
56	R = 3-pyridyl	-	1.023	-	-	-	0.012	-	-	

**Table 3:** Summary of the anti-cancerous activity of flavopiridol-based derivatives.

S. No.	Derivatives	Experimental Models	Dose (IC50) in µM	Refs.
	*cis*-5,7-dihydroxy-2-(R)-8-[4-(3-en)-piperidinyl]-1-benzopyran-4-one			[34]
1	R = 2-chlorophenyl	MCF-7	0.75	
2	R = 3-chlorophenyl	MCF-7	1	
3	R = 4-chlorophenyl	MCF-7	3	
4	R = 2-florophenyl	MCF-7	1	
5	R = 2-chlorophenyl	MCF-7	1	
6	R = 2-bromophenyl	MCF-7	1.5	
7	(3*S*,4*R*)-2-[(2-Chlorophenyl)thio]-5,7-dihydroxy-8-(3-hydroxy-1-methyl-4-piperidinyl)-4H-1-benzopyran-4-one	PC3, Mia PaCa-2, HCT116, A2780	0.02, 0.03, 0.21, 0.87	[131]
8	4,6-Dihydroxy-7-(1-methyl-piperidin-4-yl)-2-[1-phenylmeth-(*E*)-ylidene]-benzofuran-3-one	HCT-116	>50	[130]
9	2-[1-(2-Chloro-phenyl)-meth-(*E*)-ylidene]-4,6-dihydroxy-7-(1-methyl-piperidin-4-yl)-benzofuran-3-one	HCT-116	20.1	
10	4,6-Dihydroxy-7-(1-methyl-piperidin-4-yl)-2-[1-(4-nitrophenyl)-meth-(*E*)-ylidene]-benzofuran-3-one	HCT-116	35.6	
11	4-[4,6-Dihydroxy-7-(1-methyl-piperidin-4-yl)-3-oxo-3Hbenzofuran-(2*E*)-ylidenemethyl]-benzene sulfonamide	HCT-116	>50	
12	4,6-Dihydroxy-2-[1-[4-(4-methyl-piperazin-1-yl)-phenyl]-meth-(*E*)-ylidene]-7-(1-methyl-piperidin-4-yl)-benzofuran-3-one	HCT-116	24.8	
	R_1_-*N*-[2-(2-Chlorophenyl)-5,7-dihydroxy-4-oxo-4Hchromen-8-yl]-R_2_			[134]
13	R_2_ = benzamide	MCF-7	8.5	
14	R_2_ = 4-methylbenzamide	MCF-7	9.7	
15	R_2_ = 4-methoxybenzamide	MCF-7	13	
16	R_1_ = 3,5-Dichloro; R_2_ = 2-hydroxyl benzene sulfonamide	ID-8, MCF-7	24, 17	
17	2-(2-chlorophenyl)-5,7-dihydroxy-8-((2*R*,3*S*)-2-(hydroxymethyl)-1-methylpyrrolidin-3-yl)-4H-chromen-4-one hydrochloride (P-276-00)	HCT-116, T-24, U2OS, SiHa, MCF-7, PC-3, HT-29, Colo-205, Caco-2, HL-60, SW-480, H-460, MRC-5, WI-38	0.31, 0.39, 0.4, 0.42, 0.52, 0.56, 0.6, 0.65, 0.65, 0.75, 0.76, 0.8, 11.5, 16.5	[132]
18	2-(2-Chloro-4-(trifluoromethyl)phenyl)-5,7-dihydroxy-8-((2*R*,3*S*)-2-(hydroxymethyl)-1-methylpyrrolidin-3-yl)-4H-1-benzopyran-4-one (Voruciclib)	RIVA	56.3%	[135]
19	2-(2,6-dichlorophenyl)-5,7-dihydroxy-8-[(3*S*,4*R*)-3-hydroxy-1-methylpiperidin-4-yl]chromen-4-one (IIIM-290)	HL60, MOLT-4, MIAPaCa-2, Panc-1, PC-3, DU145, MCF-7, MDAMB-231, MDAMB-468, BT-549, T47D, Caco-2, SW630, Colo-205, HCT116, A549, NCIH322, NCIH522, HOP62, HOP92, NCIH-226, 786-O, A431, LOXIMVI, OVCAR-3, OVCAR-4, OVCAR-5, mouse adenocarcinoma, HGF, fR2, HEK293	0.9, 0.5, 1, 4, 6, 5, 4, 4, 4, 5, 6, 7, 0.3, 7, 5, 4, 2, 5, 7, 3, 4, 6, 8, 4, 8, 9, 7, 1.2, 18, 19, 22	[23]
20	2-(4-((1H-benzo[d]imidazol-2-yl)thio)phenyl)-5,7-dihydroxy-8-(1-methyl-1,2,3,6-tetrahydropyridin-4-yl)-4H-chromen-4-one	NCI-N87, K562, SKBR3, HCT116, SKOV3, PC3, MiaPaCa-2	0.183, 0.194, 0.254, 0.293, 0.595, 0.742, 0.852	[25]
21	5,7-Dihydroxy-8-(1-methyl-1,2,3,6-tetrahydropyridin-4-yl)-2-(4-((1-methyl-1H-benzo[d]imidazol-2-yl)thio)phenyl)-4Hchromen-4-one	HCT116, SKBR3, SKOV3, K562, SKBR3, MiaPaCa-2, NCI-N87	0.173, 0.243, 0.295, 0.300, 0.352, 0.448, 4.796	
22	5,7-Dihydroxy-8-(1-methyl-1,2,3,6-tetrahydropyridin-4-yl)-2-(4-((5-phenyl-1H-imidazol-2-yl)thio)phenyl)-4H-chromen-4-one	HCT116, SKBR3, PC3, SKOV3, K562, MiaPaCa-2, NCI-N87	0.219, 0.249, 0.276, 0.344, 0.345, 0.361, 0.391	
23	2-(4-((1H-benzo[d]imidazol-2-yl)thio)-2-chlorophenyl)-5,7-dihydroxy-8-(1-methyl-1,2,3,6-tetrahydropyridin-4-yl)-4H-chromen-4-one	NCI-N87, K562, SKBR3, HCT116, PC3, SKOV3, MiaPaCa-2	0.012, 0.036, 0.066, 0.170, 0.326, 0.333, 0.361	
24	2-(2-Chloro-4-((1-methyl-1H benzo[d]imidazol-2-yl)thio) phenyl)-5,7-dihydroxy-8-(1-methyl-1,2,3,6-tetrahydropyridin-4-yl)-4H-chromen-4-one	NCI-N87, K562, MiaPaCa-2, SKBR3, PC3, SKOV3, HCT116	0.049, 0.051, 0.053, 0.054, 0.085, 0.094, 0.181	
	2-R-5,7-dihydroxy-8[(3*S*,4*R*)-3-hydroxy-1-methylpiperidin-4-yl]chromen-4-one			[133]
25	R = phenyl	HeLa	0.190	
26	R = 3-chlorophenyl	HeLa	0.170	
27	R = 4-chlorophenyl	HeLa	0.200	
28	R = 2-fluorophenyl	HeLa	0.274	
29	R = 4-fluorophenyl	HeLa	0.200	
30	R = 4-bromophenyl	HeLa	0.280	
31	R = 4-tert-butylphenyl	HeLa	0.660	
32	R = 4-trifluoromethylphenyl	HeLa	1.200	
33	R = 4-hydroxyphenyl	HeLa	20.920	
34	R = 2-pyridyl	HeLa	1.490	
35	R = 3-pyridyl	HeLa	3.300	
36	R = 4-pyridyl	HeLa	3.350	
37	R = 2-chloro-3-pyridyl	HeLa	0.177	
38	R = 5-methylisoxazole	HeLa	0.645	
39	R = 3-vinylphenyl	HeLa	0.195	
40	R = 4-vinylphenyl	HeLa	0.219	
41	R = 4-fluorophenyl	HeLa	0.266	
42	R = 2-bromophenyl	HeLa	0.331	
43	R = 3-pyridyl	HeLa	1.440	

**Table 4:** Various clinical trials are reported for the inhibition of tumor development and metastasis.

Clinical Trial	Cancer	Phase	Status	Sample Size	Treatment
NCT00003256	Recurrent prostate cancer	Phase II	Completed	40	FP
NCT00007917	Adult solid tumors	Phase I	Completed	58	FP and gemcitabine hydrochloride
NCT00016939	Renal cell carcinoma	Phase II	Completed	35	FP
NCT00070239	Hematopoietic, adult solid tumors, and lymphoid cancer	Phase I	Terminated	100	FP and fludeoxyglucose F 18
NCT00006245	Metastatic esophageal cancer	Phase II	Completed	37	FP and paclitaxel
NCT00006485	Adult solid tumors	Phase I	Completed	50	FP and irinotecan hydrochloride
NCT00112684	Metastatic solid tumors	Phase I	Terminated	25	FP
NCT00324480	Adult solid tumors	Phase I	Completed	60	FP and vorinostat
NCT00020332	Metastatic breast cancer	Phase I/II	Completed	49	FP and docetaxel
NCT00331682	Recurrent pancreatic cancer and pancreatic adenocarcinoma	Phase II	Completed	10	FP and docetaxel
NCT00080990	Adult solid tumors	Phase I	Completed	46	FP, fluorouracil, oxaliplatin, and leucovorin calcium
NCT00039455	Metastatic breast cancer	Phase I	Terminated	50	FP and trastuzumab
NCT00087282	Advanced liver cancer	Phase II	Completed	32	FP and irinotecan hydrochloride
NCT00072436	Adult solid tumors	Phase I	Completed	58	FP and gemcitabine hydrochloride
NCT00045448	Advanced solid tumors	Phase I	Completed	56	FP and docetaxel
NCT00046917	Adult solid tumors	Phase I	Completed	13	FP, irinotecan hydrochloride, and cisplatin
NCT00019344	Adult solid tumors, lymphoma, prostate cancer, and small intestine cancer	Phase I	Completed	36	FP
NCT00047307	Locally advanced and unresectable pancreatic cancer	Phase I	Completed	46	FP, gemcitabine hydrochloride, and radiation therapy
NCT00023894	Recurrent or persistent endometrial cancer	Phase II	Completed	51	FP
NCT00016185	Advanced solid tumors	Phase I	Completed	24	FP and docetaxel
NCT00003690	Breast cancer, melanoma, prostate cancer, and adult solid tumors	Phase I	Completed	48	FP
NCT00042874	Metastatic solid tumors	Phase I	Completed	77	FP, irinotecan hydrochloride, fluorouracil, and leucovorin calcium
NCT00003004	Refractory or recurrent solid tumors	Phase I	Completed	73	FP, cisplatin, and paclitaxel
NCT00079352	Metastatic solid tumors	Phase I	Completed	24	FP, gemcitabine hydrochloride, and irinotecan hydrochloride
NCT00020189	Metastatic head and neck cancer, Thromboembolism	Phase II	Completed	37	FP, acetylsalicylic acid, and clopidogrel bisulfate
NCT00094978	Small cell carcinoma, non-small cell lung cancer, esophageal neoplasms, and mesothelioma	Phase I	Terminated	23	FP and depsipeptide
NCT00021073	Unspecified adult solid tumors	Phase I	Completed	90	FP, leucovorin calcium, fluorouracil, and irinotecan hydrochloride
NCT00012181	Recurrent childhood solid tumors, recurrent neuroblastoma, recurrent osteosarcoma, and recurrent retinoblastoma	Phase I	Completed	30	FP
NCT00957905	Recurrent or relapsed germ cell tumors	Phase II	Completed	36	FP, leucovorin calcium, fluorouracil, and oxaliplatin
NCT00064285	Leukemia	Phase I	Completed	22	FP and imatinib mesylate
NCT00991952	Advanced stomach and gastroesophageal junction cancer	Phase II	Completed	19	FP and irinotecan hydrochloride
NCT00083122	Advanced ovarian epithelial cancer and primary peritoneal cancer	Phase II	Completed	45	FP and cisplatin
NCT00082784	Recurrent or refractory indolent B-cell neoplasms	Phase I	Completed	93	FP and bortezomib
NCT00098579	Metastatic or recurrent sarcoma	Phase I	Completed	36	FP and doxorubicin hydrochloride
NCT03441555	Relapsed or refractory acute myeloid leukemia	Phase I	Completed	36	FP and venetoclax
NCT00047203	Relapsed or refractory multiple myeloma	Phase II	Completed	35	FP
NCT03298984	Acute myeloid leukemia	Phase I	Completed	32	FP, cytarabine, and daunorubicin
NCT03969420	Acute myeloid leukemia	Phase II	Terminated	11	FP and cytarabine
NCT03563560	Acute myeloid leukemia	Phase I	Completed	10	FP, cytarabine, mitoxantrone, and daunorubicin
NCT00005974	Recurrent and metastatic soft tissue sarcoma	Phase II	Completed	18	FP
NCT02520011	Acute myeloid leukemia	Phase II	Terminated	104	FP, cytarabine, and mitoxantrone
NCT03593915	Myelodysplastic syndromes	Phase I/II	Terminated	20	FP and decitabine or azacitidine
NCT00470197	Relapsed or refractory acute leukemia	Phase I	Completed	35	FP, cytarabine, and mitoxantrone hydrochloride
NCT00112723	Relapsed or refractory lymphoma and multiple myeloma	Phase I/II	Terminated	46	FP
NCT00445341	Relapsed mantle cell lymphoma and diffuse large B-cell lymphoma	Phase I/II	Completed	28	FP
NCT01349972	Acute myeloid leukemia	Phase II	Completed	172	FP, mitoxantrone hydrochloride, cytarabine, and daunorubicin hydrochloride
NCT00795002	Acute myeloid leukemia	Phase II	Completed	78	FP, mitoxantrone hydrochloride, and cytarabine
NCT00634244	Relapsed or refractory acute myeloid leukemia	Phase II	Completed	92	FP, mitoxantrone hydrochloride, carboplatin, cytarabine, sirolimus, etoposide, and topotecan hydrochloride
NCT00003039	Lymphoma	Phase II	Completed	40	FP
NCT00058240	Chronic lymphocytic leukemia and lymphocytic lymphoma	Phase I/II	Completed	52	FP
NCT00005971	Metastatic malignant melanoma	Phase II	Completed	17	FP
NCT00101231	Relapsed or refractory acute myeloid leukemia, acute lymphoblastic leukemia, and chronic myelogenous leukemia	Phase I	Terminated	88	FP
NCT00058227	Lymphoproliferative disorders or mantle cell lymphoma	Phase I	Completed	37	FP, fludarabine phosphate, and rituximab
NCT00098371	Chronic lymphocytic leukemia and prolymphocytic leukemia	Phase II	Terminated	64	FP
NCT00003620	Chronic lymphocytic leukemia	Phase II	Completed	37	FP
NCT00464633	Chronic lymphocytic leukemia	Phase II	Completed	165	FP
NCT00278330	Relapsed or refractory acute leukemia, chronic myelogenous leukemia, and refractory anemia	Phase I	Completed	24	FP and vorinostat
NCT00735930	Relapsed or refractory B-cell chronic lymphocytic leukemia and small lymphocytic lymphoma	Phase I	Completed	39	FP and lenalidomide
NCT00377104	B-cell chronic lymphocytic leukemia and small lymphocytic lymphoma	Phase I	Terminated	24	FP
NCT00016016	Acute leukemia	Phase I/II	Completed	53	FP, cytarabine, and mitoxantrone hydrochloride
NCT01076556	B-cell chronic lymphocytic leukemia and small lymphocytic lymphoma	Phase I	Terminated	9	FP, cyclophosphamide, and rituximab
NCT00407966	Acute myeloid leukemia	Phase II	Completed	45	FP, cytarabine, and mitoxantrone hydrochloride
NCT00005074	Relapsed or untreated mantle cell lymphoma	Phase II	Completed	33	FP

## Data Availability

This document includes citations for all the data that were analyzed throughout the literature review.
